# Hallmarks of DNA Damage Response in Germination Across Model and Crop Species

**DOI:** 10.3390/genes16010095

**Published:** 2025-01-17

**Authors:** Federico Sincinelli, Shraddha Shridhar Gaonkar, Sri Amarnadh Gupta Tondepu, Conrado Jr Dueñas, Andrea Pagano

**Affiliations:** Department of Biology and Biotechnology ‘L. Spallanzani’, University of Pavia, via Ferrata 9, 27100 Pavia, Italy

**Keywords:** DNA damage response, DNA repair pathways, pre-germinative metabolism, hallmark, seed priming, abiotic stress

## Abstract

DNA damage response (DDR) contributes to seed quality by guarding genome integrity in the delicate phases of pre- and post-germination. As a key determinant of stress tolerance and resilience, DDR has notable implications on the wider scale of the agroecosystems challenged by harsh climatic events. The present review focuses on the existing and documented links that interconnect DDR efficiency with an array of molecular hallmarks with biochemical, molecular, and physiological valence within the seed metabolic networks. The expression of genes encoding DDR sensors, transducers, mediators, and effectors is interpreted as a source of conserved hallmarks, along with markers of oxidative damage reflecting the seed’s ability to germinate. Similarly, the accumulation patterns of proteins and metabolites that contribute to DNA stability are predictive of seed quality traits. While a list of candidates is presented from multiple models and crop species, their interaction with chromatin dynamics, cell cycle progression, and hormonal regulation provides further levels of analysis to investigate the seed stress response holistically. The identification of novel hallmarks of DDR in seeds constitutes a framework to prompt validation with different experimental systems, to refine the current models of pre-germinative metabolism, and to promote targeted approaches for seed quality evaluation.

## 1. Introduction

DNA repair is a major contributor to seed vigor, along with the array of molecular processes that must coordinate to ensure seed viability, germination, and seedling establishment. This interplay represents a source of possible markers for crop improvement through breeding, biotechnology, and seed vigorization protocols [1,2]. DNA is exposed to a diverse array of lesions that can be classified based on their cause. UV or ionizing radiation causes strand crosslinks, dimers, and strand breaks, whereas alkylating agents result in methylated bases. Reactive oxygen species (ROS) accumulation causes base oxidation. Errors during replication result in mismatches and DNA-protein adducts. Moreover, apurinic sites and mismatches can also arise from spontaneous deamination [3,4]. The susceptibility to DNA damage has determined the evolution of an equally complex array of molecular processes, globally named DNA damage response (DDR), aimed at sensing and repairing such alterations. DDR is also able to integrate repair processes within the wider regulatory network of cell proliferation and death, with implications on phenotypic plasticity and developmental processes from seed to seedling [1,2].

The general organization of the DDR cascade is conserved between plants and animals and encompasses a set of components, including DNA damage sensors, signal transducers, mediators, and effectors [4,5,6], that can be summarized as follows.

DNA damage sensing. The sensing function is performed by protein complexes such as MRN (MRE11, meiotic recombination 11; RAD50, radiation sensitive 50; NBS1, Nijmegen breakage syndrome 1), recognizing DSBs (double-strand breaks), and RPA (replication protein A), associated with SSBs (single-strand breaks) [7,8].

DDR signal transduction. The next layer of DDR involves the major signal transducers ATM (ataxia-telangiectasia mutated) and ATR (ataxia telangiectasia and Rad3-related protein) kinases [9], responsible for the signal cascades eliciting DDR activation [10]. Despite the similar function, ATM responds to DSBs whilst ATR responds to SSBs [11]. The phosphorylated histone variant γH2AX is also involved in DSB signaling [12].

DDR Regulation. Both ATM and ATR signaling cascades convey to the transcriptional factor SOG1 (suppressor of the γ response 1), considered the master regulator of plant DDR as a functional homolog of the vertebrate p53 protein [12,13]. SOG1 is a member of the NAC domain family (NAM, no apical meristem; ATAF1, *Arabidopsis thaliana* activation factor; CUC2, cup-shaped cotyledon), originally characterized based on the suppression of the γ irradiation response in *sog1* Arabidopsis mutants. In the absence of the SOG1 function, cell cycle progression is observed, together with increased susceptibility to irradiation and impairments in major developmental programs, including seed-to-seedling transition [14,15]. SOG1 homologs have been identified in *Oryza sativa*, *Zea mays*, *Glycine max*, *Medicago truncatula*, and other species [8]. In some cases, such as *M. truncatula*, *G. max*, and *Populus trichocarpa*, multiple copies of the *SOG1* gene were detected, suggesting redundant functions based on expression patterns [16].

Responses mediated by SOG1. Models of SOG1-dependent transcriptomic dynamics have been obtained through DREM (Dynamic Regulatory Event Miner) in Arabidopsis seedlings following γ-irradiation, highlighting ~2400 responsive genes with biological functions ranging from DNA repair, cell cycle control, immunity, ROS detoxification, and programmed cell death [17]. These different outcomes are determined by the DDR machinery based on the severity of DNA lesions. Despite this general organization, the signaling that links SOG1 to the expression of DNA repair genes is still under investigation, considering also the recent characterization of SOG1-independent pathways [18].

DNA repair pathways. DNA repair comprises an array of enzymes able to process DNA lesions. For example, SSBs, as well as base oxidation, alkylation, and deamination, are repaired via base excision repair (BER). DNA lesions such as bulky helix distortion are processed through nucleotide excision repair (NER). Dimers and alkylation are resolved via direct reversal repair (DRR). Mismatches are repaired via the specific mismatch repair (MMR) mechanism. DSBs are repaired via homologous recombination (HR) or nonhomologous end joining (NHEJ) [3,4,19,20]. The repair of DNA-topoisomerase crosslinks arising from replicative errors is performed by TDP1 (tyrosyl DNA-phosphodiesterase) proteins [21,22,23].

Cell cycle regulation. SOG1 also represents the connection between DDR and cell cycle arrest [12]. The integration of parallel signaling cascades regulates the cell cycle along with repair processes to allow the degree of phenotypic plasticity necessary to repair, resist, or survive different DNA lesions. Specifically, in the presence of DSBs, ATM, and SOG1 activate cyclin-dependent kinase inhibitors that down-regulate genes involved in the G2/M transition. Otherwise, in response to replicative stress, ATR and SOG1 activate WEE1, a kinase that blocks the cell cycle, thus suggesting that the cell cycle is selectively arrested based on damage type [4,17,24,25]. Meta-analyses of transcriptomic datasets have led to the identification of cyclin-dependent kinase inhibitors that are responsive to ATM-SOG1 signaling in the presence of ROS accumulation, integrating the control of cell-cycle progression with the responsiveness to oxidative damage [26]. Besides cell cycle arrest, SOG1-mediated response to DSBs can also result in endoreduplication, preventing the proliferation of cells with damaged DNA without compromising tissue functionality [27].

Programmed cell death (PCD). Finally, in the cases when DNA damage reaches critical thresholds, SOG1 also promotes PCD [13,15]. For example, UV and ionizing radiation induce PCD in Arabidopsis stem cells via ATM or ATR and SOG1 [28].

This overview of DDR in plants, considering the interaction with key cellular processes and their implication on development and stress response, constitutes a premise to explore the specific role of DDR in the context of seed pre-germinative metabolism. This review adopts a comparative approach to highlight convergent and divergent features, identifying recurrent and reliable determinants of genome stability in germinating seeds in crop, model, and wild species based on the available literature. Different levels of analysis are considered, including gene expression patterns, protein and metabolite accumulation, chromatin dynamics, and epigenetic modifications, to highlight methodological constrains and promote holistic interpretations. The molecular players directly or indirectly linked to DDR activation in seeds are interpreted as seed quality hallmarks, useful to understand pre-germinative metabolism from the perspective of genome maintenance under a variety of environmental conditions.

These premises are developed along the hypothesis that DDR-related hallmarks in seed metabolism can be identified, validated, and applied. Particularly, this hypothesis will be explored through the following questions:

(1) What defines an optimal DDR-related hallmark in seed metabolism?
(2) Which theoretical and experimental constraints limit the identification of DDR-related hallmarks?
(3) Can DDR-related hallmarks be applied to investigate and improve seed quality?

## 2. DNA Damage Is a Challenge for Seed Metabolism and Germination Performance

Climate change, as well as the growing demand for food, requires higher agricultural yields and stress-tolerant crops, with increased water efficiency and reduced use of chemical fertilizers and pesticides. Seed germination represents the first step of a plant life cycle, and any suboptimal parameter results in poor development, ultimately affecting yields. Therefore, investigating the dynamics occurring before the radicle protrusion stage, namely the pre-germinative metabolism, is crucial to understanding the molecular and physiological bases of a successful seedling establishment and an efficient stress tolerance [2,29].

Compared to seedlings and adult plants, mature orthodox seeds can survive a range of extreme conditions, including heat, freezing cold, desiccation, and prolonged storage. Suboptimal storage conditions lead to the accumulation of DNA damage due to the occurrence of non-enzymatic, spontaneous reactions that induce oxidation of biological molecules, including DNA [30,31,32]. When a seed is quiescent, protective mechanisms such as intracellular glassy state, antioxidant compounds, and osmoprotectants represent a defense against oxidative damage. On the other hand, during and after germination, these mechanisms are complemented by antioxidant enzymes and active repair processes [33]. DDR activation, crucial for removing the damage accumulated before and during germination, requires the arrest of cell cycle progression. Thus, when DDR components are defective, germination is delayed, seedling growth is impaired, and stress tolerance is compromised [2,32]. Seeds experience both endogenous and exogenous threats to DNA integrity. For example, oxidation during storage can damage genome and cell structures, while the reactivation of seed metabolism before germination increases the endogenous production of ROS from lipid catabolism in peroxisomes and from the respiratory chain in mitochondria and plastids. The high oxidative potential of ROS induces several types of DNA lesions, including base oxidation and strand breaks, although controlled ROS levels play key roles in stress signaling and cell wall plasticity during radicle elongation [30]. Additionally, exogenous stressors such as temperature, salinity, drought, flooding, soil contaminants, infections, etc., exacerbate ROS accumulation, DNA damage, and developmental defects. For example, soil contamination with heavy metals (including Ni, Cu, Zn, Cd, and Pb) impairs germination by causing genotoxic stress, chromosomal aberrations, and reduced meristem activity [34].

In the wider context of seed physiology, ABA (abscisic acid), counterbalanced by GAs (gibberellins), plays several roles in seed metabolism by promoting stress responses over germination, indirectly contributing to genome stability. ABA signaling stimulates the accumulation of antioxidant compounds, LEA (late embryogenesis abundant) proteins, HS (heat shock) proteins, and other players with protective functions [33]. Moreover, direct links between hormonal signaling and DDR have been reviewed [35].

**Figure 1:**
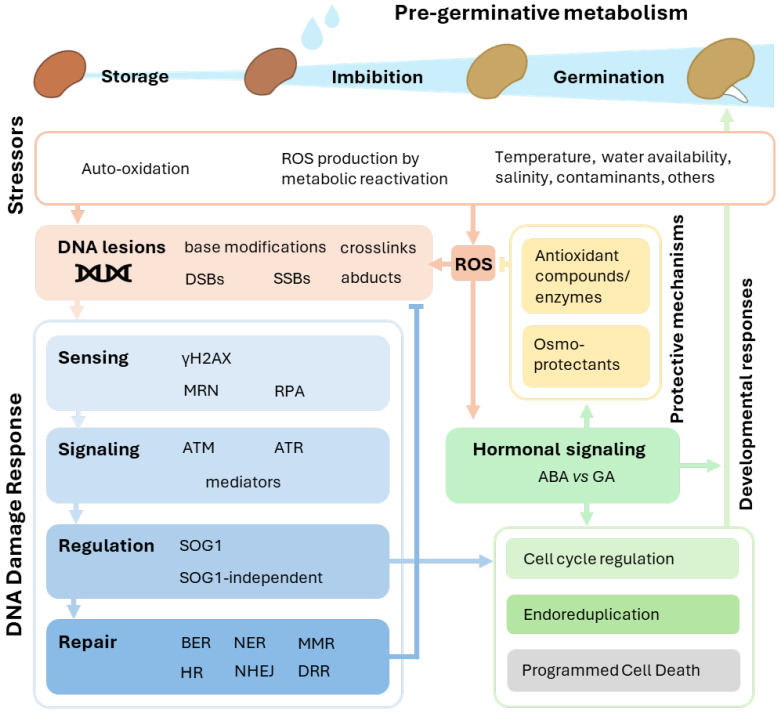
Overview of DDR in the context of seed pre-germinative metabolism. Phases of pre-germinative metabolism, associated stressors, DNA lesions, interactions with other seed protective mechanisms, and DDR implications on the cell cycle are reported. MRN, MRE11 (meiotic recombination 11) RAD50 (radiation sensitive 50) NBS1 (Nijmegen breakage syndrome 1); DSB, double-strand breaks; SSBs, single-strand breaks; RPA, replication protein A; ATM, ataxia-telangiectasia mutated; ATR, ataxia telangiectasia and Rad3-related protein; γH2AX, phosphorylated histone H2AX; SOG1, suppressor of the γ response 1; BER, base excision repair; NER, nucleotide excision repair; MMR, mismatch repair; HR, homologous recombination; NHEJ, nonhomologous end-joining; DRR, direct reversal re-pair; ABA, abscisic acid; Gas, gibberellins; ROS, reactive oxygen species.

The pervasive nature of DDR, as a determinant of germination progression and stress tolerance in seed metabolism, can be explored by examining the DDR features and interconnections with other cellular processes. This represents a promising route to identify the key molecular factors that drive successful germination, as exemplified in Figure 1.

## 3. Criteria to Define a Molecular Hallmark in Seed Metabolism

This review defines a “hallmark of seed quality” as any gene, protein, epigenetic modification, chromatin feature, metabolite, or other factors whose accumulation or depletion patterns can be correlated to, or be predictive of, successful/unsuccessful germination, seedling establishment, stress tolerance profile, or responsiveness to vigorization protocols. With these premises, a DDR-related hallmark can be a DDR player itself, a factor regulating DDR, or a molecule that acts as a proxy of DDR efficiency. Moreover, an effective hallmark should fulfill certain requirements, namely universality, reliability, detectability, and applicability.

Universality. The first requirement for an ideal hallmark is universality, defined as a conserved predictive power across species, both close and far in evolutionary terms. Comparative studies have highlighted a high degree of conservation in the upstream DDR players, namely sensing and signaling components, as well as DNA repair enzymes. Conversely, higher functional diversity characterizes specific DDR components in plants adapted to extreme environments, especially when comparing ancient plants, monocots, and dicots [5,36]. *OGG1* (*8-Oxoguanine DNA glycosylase 1*) gene expression patterns in seeds responding to oxidative stress can be indicated as an example of universality [37,38,39,40]. On the other hand, a hallmark can be functional in a taxon, a species, or a cultivar but not in others, thus compromising universality. This is especially true with DDR players that are multifunctional or operate major regulatory switches between different transcriptional programs and diverging outcomes in seed metabolism. For example, evolutionary divergent features can occur in the control of the cell cycle to allow DNA repair. In plants, the G2/M transition in the cell cycle is mediated by the cyclin-dependent kinase CDKB2. In Arabidopsis, DNA damage induces CDKB2 degradation to stop the cell cycle and allow DNA repair, thus promoting endoreduplication over mitosis and increasing the fraction of polyploid cells. Conversely, in rice (*O. sativa*) plants, DNA damage induces the accumulation of the CDKB2 ortholog, endoreduplication is not promoted over cell cycle arrest, and polyploid cells are mostly limited to specific tissues (i.e., endosperm) [41]. These observations highlight the need for exploring the relative roles of DDR players in different species and contexts to refine their uses as universal DDR hallmarks in germination. Broadly speaking, the comparative perspective necessary to find universal hallmarks needs to face the heterogeneity of experimental approaches applied to different models, species, and experimental systems. This represents an intrinsic limitation for universality, indicating the necessity and the potential of data mining to identify conserved trends across species and experimental conditions.

Reliability. The reliability of a hallmark correlates with experimental reproducibility and validation in a diverse array of experimental systems, corroborated by extensive data mining. Moreover, defining the temporal timeframes of DNA damage sensing, response and repair can be relevant to obtain mechanistic explanations of stress response and to define the applicability ranges of DDR-related hallmarks. For example, SOG1 activates the expression of hundreds of DDR genes following γ-irradiation in Arabidopsis seeds and seedlings with timeframes ranging from minutes to hours [12,17], thus allowing more refined models of transcriptional activation. In rice seeds, DDR activation is reported within 48 h following γ-irradiation [42]. The transitions from storage to pre-germination and from pre-germination to seedling establishment imply relevant considerations to identify DDR hallmarks. For example, prolonged storage induces DSBs that impair germination performance. In this context, ATM and SMR kinases control replication and delay germination in the presence of damage following seed aging to allow repair. Conversely, Arabidopsis *atm* mutants germinate faster but with an increased occurrence of chromosomal defects [31]. Divergent features can be pinpointed between seeds and seedlings concerning the choice between repair and cell death as mediated by SOG1. Compared with seedlings, seeds display higher resistance to DNA damage, reduced cell cycle activity, and a delayed PCD. This can be interpreted as a concerted action to repair damage accumulated in seeds during aging while preparing for radicle protrusion, as highlighted through comparative gene expression analysis in Arabidopsis DDR mutants [32]. Defining cell- and tissue-specificity is also relevant for assessing the applicability of DDR-hallmarks. As an example, SOG1 induces cell death in response to DNA lesions in mitotically compromised meristematic stem cells and their immediate descendants to prevent aberrations from spreading while maintaining overall tissue functionality [43]. The contribution of other factors, besides repair processes, must be considered to contextualize the applicability of a hallmark. Indeed, the metabolically inactive and maternally derived protective layers constituting the seed coat contribute to seed longevity, as shown in Arabidopsis mutants with altered seed coat structure [33].

Detectability. The availability of methods for rapid hallmark detection/quantification is crucial for large-scale seed quality evaluation. However, hallmarks whose assessment is time- or resource-consuming could still be used for biotechnological purposes, marker-assisted breeding programs, or for increasingly detailed modeling of seed metabolism [2,44,45]. In this context, the study of seed exudates and volatile organic compounds (VOCs) represents a promising route for a rapid and non-invasive assessment of seed quality and stress status. VOC analysis has been applied to detect deterioration in rice grains following fungi and insect infection [46]. Nonetheless, VOC detection from seeds has unexpressed potential when associated with molecular models of seed metabolism reacting to stress, considering the footprints of (e.g.,) ROS accumulation and DNA damage on VOC composition [47]. Detectability needs to be complemented with instrument sensitivity and statistical soundness. When interpreting the changes in the accumulation of a specific hallmark, determining a threshold of biological significance can be challenging and must be defined according to the specific nature of the hallmark. For example, DNA repair enzymes that operate downstream in DDR signaling are expected to be proportionally accumulated to fulfill specific functions. Conversely, signal transducers and master regulators might be more likely to fulfill multifaceted functions based on small variations amplified by signaling cascades. This represents an intrinsic complication in the analysis and interpretation of high-throughput datasets because uniform thresholds of statistical significance might conceal biologically relevant variations while evidencing inconsequential ones.

Applicability. When considering practical outcomes of DDR-related hallmarks for farming and germplasm banks, specific distinctions can be made to explore their potential for seed quality assessment and improvement. Concerning seed quality assessment, certain hallmarks could be predictive of deterioration, germination performance, and stress tolerance. The mentioned volatilomic approaches are promising in this sense for their low invasiveness when compared with genomic, transcriptomic, proteomic, and metabolomic approaches [46,47] that, conversely, offer much wider datasets for hallmark screening. Concerning seed quality improvement, metabolites or compounds contributing to DNA integrity could be used as nutrients, seed priming agents, or seed coating additives, whereas controlled stressors can enhance resilience [1,2]. DDR genes, proteins, and chromatin modifications with significant impact on stress tolerance profiles can have unexpressed potential in biotechnological approaches and marker-assisted breeding aiming at improving germination and seedling establishment under a variety of environments [2,44]. Regardless of its direct applicability for seed quality assessment and improvement, a DDR-related hallmark can contribute to the current understanding of seed metabolism and stress tolerance in more theoretical ways by highlighting regulatory mechanisms or interactions with other stress tolerance strategies (e.g., antioxidant machinery, stress memory) [2,44,45].

**Figure 2:**
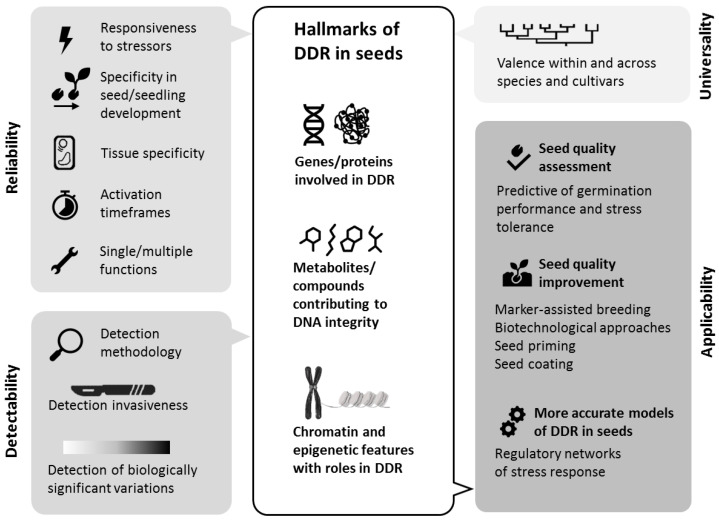
Outline of the proposed criteria for the definition and identification of DDR-related hallmarks in seed metabolism in relation to possible applicative outcomes for seed technology, summarizing key aspects contributing to universality, reliability, detectability, and applicability.

Hallmarks embodying the required criteria of universality, reliability, detectability, and applicability are challenging to identify and validate. However, these criteria can guide researchers and industries toward both applicative and theoretical outcomes, as outlined in Figure 2.

## 4. Expression Patterns of DDR Genes as a Reproducible Hallmark of Seed Quality

When searching for DDR hallmarks, the deepest level of complexity is related to the presence, number, and expression of genes involved in DDR processes. The expression of DDR sensors, transducers, mediators, and effectors was evaluated during germination. For instance, the expression of genes encoding the components of the sensor complex MRN was upregulated in Arabidopsis seeds upon ABA administration that caused ROS accumulation and DNA damage [48]. Upregulation of the same genes occurred in *M. truncatula* seeds exposed to hydropriming and desiccation stress [49]. Several studies corroborate the crucial roles of ATR and ATM in seed DDR. For example, *ATM* and *ATR* are upregulated in Arabidopsis seeds subjected to fluctuating temperature and hydration, and *atm* mutant seeds display increased damage and lower survival rates [31].

*SOG1* gene expression in seeds is required to regulate root development when DNA lesions are present [50]. The existence of a small gene family encoding SOG1 orthologs was evidenced in several species, including *G. max*, *M. truncatula*, *P. trichocarpa*, and *Kalankoe fedtschenkoi*. In particular, in *M. truncatula*, the expression of two *SOG1* genes was monitored during imbibition, hydropriming, and overpriming (prolonged priming with loss of desiccation tolerance), highlighting upregulation during imbibition but different responsiveness to overpriming [16,49].

The expression of genes encoding enzymes actively repairing DNA lesions, modification of DNA nucleotides, and other types of alterations, collectively known as effectors, was also considered. The most relevant data were obtained from BER genes. One of the most represented genes in terms of species and treatments is *OGG1*, responsible for the repair of 7,8-dihydro-8-oxoguanine lesions [51,52]. This type of lesion is considered a universal indicator of DNA damage in both plants and animals, and commercial kits for the immunochemical detection of this lesion are available. For instance, ELISA (enzyme-linked immunosorbent assay) was applied to detect the increased levels of 7,8-dihydro-8-oxoguanine in *M. truncatula* seeds responding to osmotic stress [21], and in *Acer pseudoplatanus* seeds subjected to aging [53]. *OGG1* gene overexpression resulted in a better germination performance in Arabidopsis seeds exposed to methyl viologen, NaCl, and mannitol [38]. *OGG1* orthologs were upregulated in *M. truncatula* seeds exposed to sodium butyrate-inducing DNA damage [54], in hydroprimed eggplant (*Solanum melongena*) seeds [45], and in eggplant seeds exposed to NaCl [55]. *FPG* genes encoding formamido-pyrimidine-DNA-glycosylase involved in the first step of BER were investigated in multiple species, including Arabidopsis [56], *M. truncatula* [37,54], and eggplant [45], showing upregulation during imbibition in physiological and stress conditions. The *MBD4L* (*methyl-CpG-binding domain protein 4 like*) gene, encoding a BER DNA glycosylase, is upregulated in imbibed aged seeds, and *mbd4l-1* arabidopsis mutants display impaired germination [57]. Moreover, comparative studies of *FPG* and *OGG1* gene expression in wheat (*Triticum aestivum*), barley (*Hordeum vulgare*), and rye (*Secale cereale*) during germination have highlighted divergent expression patterns [40]. The *ALKBH1* (*alkylation repair homolog*) gene, coding for a BER enzyme that removes alkylated bases, was upregulated in *M. truncatula* seeds in the presence of DNA damage induced by sodium butyrate [54].

Homologous Recombination (HR) mechanisms represent another source of DDR hallmarks. For instance, *RAD51* (*radiation sensitive 1*) and *BRCA1* (*breast cancer 1*) plant homologues were upregulated in Arabidopsis seeds exposed to ABA-induced DNA damage [31,48].

The contribution of nonhomologous end joining (NHEJ) to seed DDR genes was investigated by monitoring the expression of the genes encoding DNA ligases. In Arabidopsis, *LIG1* [38] and *LIGIV* [48] genes were upregulated during imbibition and under ABA-induced DNA damage, respectively. The *LIGIV* gene was also upregulated in *M. truncatula* seeds under genotoxic stress [58]. Additionally, Arabidopsis *atlig6* mutant seeds showed higher sensitivity to cold and oxidative stress after aging [59]. Several other DDR genes have been studied in Arabidopsis seeds, namely *RAD51-54* (*radiation sensitive*, involved in HR), *Ku70-80* (involved in NHEJ), *XRCC4* (*X-ray repair cross-complementing*, involved in NHEJ), and found to be responsive to strand breaks induced by ABA treatments during imbibition [48]. Nevertheless, their role deserves investigation in other species under different experimental conditions to evaluate their potential as DDR hallmarks. This is the case of imbibed eggplant seeds responding to different salt (NaCl) concentrations and displaying upregulation of several DDR genes, including *KU70* and *MSH2* (*mutS homolog* 2, involved in MMR), *OGG1*, *NBS1* (*Nijmegen breakage syndrome 1*, involved in damage sensing), and *PCNA* (*proliferating cell nuclear antigen*, involved in replication and DNA repair recruitment) [55]. Following γ-irradiation on rice seeds, the differentially expressed DDR genes included orthologs of Arabidopsis *RecA* (*recombination A*, involved in recombination repair), *UDG* (*uracil-DNA glycosylase*, involved in base-excision in mitochondria), *PARP2*, *PARP3* (*poly ADP-ribose polymerase*, involved in Ku-independent NHEJ), and *ATR* [42]. *Z. mays* seeds exposed to camptothecin (CPT, inhibitor of topoisomerase I, inducing DSBs and cell death) showed changes in DDR gene expression, including *RNR1* (*ribonucleotide reductase*, an enzyme providing dNTPs for DNA repair), the ortholog of *RAD51* (involved in HR), *XRI-1* (X-ray induced, involved in HR), ref. [60].

Transcriptional factors responding to ABA signaling can be a source of hallmarks of the crosstalk between DDR and hormonal signaling. In this sense, OFPs (ovate family proteins) are a family of plant-specific transcriptional factors involved in DNA repair, ROS homeostasis, and hormonal response, including the GA and ABA balance that controls seed germination and radicle growth, as highlighted by Chip-Seq studies in Arabidopsis [61]. Other examples include NAC103, which positively regulates several ABA-responsive DDR genes in Arabidopsis seeds and seedlings [62], and the transcriptional factor ABI3 (abscisic acid insensitive 3), crucial for seed dormancy and longevity [33]. Overexpression of genes encoding the transcriptional factors TERF1 (tomato ethylene responsive factor 1) promotes *Nicotiana tabacum* germination in the darkness while repressing DNA repair genes [63].

Several DDR gene transcripts undergo alternative splicing in Arabidopsis (e.g., the genes encoding FPG, RAD1, UHV1, POLH enzymes) and in rice (e.g., the genes encoding class II DNA photolyase, endonuclease MUS81, and checkpoint protein RAD9) [64]. Another example is the DRT111 (DNA-damage repair toleration 111) protein, a splicing factor controlling the splicing of genes involved in ABA sensitivity and osmotic stress response, such that Arabidopsis *drt111* mutants are hypersensitive to ABA during germination [65]. Despite the evidence provided in animal models, the extent to which alternative splicing impacts DDR efficiency still needs to be systematically investigated in plants.

Besides the mentioned changes in the expression levels of protein-coding transcripts, the differential accumulation of rRNA and miRNA can be relevant as DDR-related hallmarks, with few examples currently available. Significant changes in rRNA metabolism, including the abundance of rRNA mature and precursor forms, altered nucleolar morphology, and the expression of genes involved in RNA processing were detected in the presence of DNA damage and ROS accumulation in *M. truncatula* seeds responding to dehydration [16]. Regulation operated by miRNA on DDR genes was evidenced in wheat (*T. aestivum*) seeds subjected to γ-irradiation, causing ROS and DNA damage accumulation. A negative correlation was detected between the expression of miRNAtae-miR5086 and *RAD50* gene, while other miRNAs (tae-miR164, tae-miR5086, and tae-miR1122) and DDR genes (*MRE11*, *NBS1*, *ATM*, *BRCA1*, *TDP1*, and *OGG1*) were also modulated by irradiation treatments [66].

## 5. Strategies to Investigate DDR Genes in Plant Systems with Limited Availability of Mutants

Despite the recent technological advances, the study of gene expression-based hallmarks still has important drawbacks, such as the limited number of DDR mutants available for basic and applied research along with the fact that many DDR players and pathways are not fully characterized in plants. Excluding *A. thaliana*, *O. sativa*, and a few other model plants, extensive collections of DDR mutant lines are generally not available [67,68]. Additionally, due to the involvement of DDR genes in fundamental processes for the plant life cycle, DDR mutants often display impaired viability and reproduction, thus limiting the use of mutant-based approaches.

To overcome the unavailability of DDR mutants, it is possible to treat seeds with a panel of genotoxic agents to induce DNA damage and identify those genes that participate in that specific context based on changes in their expression level. Such agents directly induce DNA damage through physical or chemical treatments that simulate an environmental stressor damaging DNA (e.g., heat, cold, salinity, drought, etc.), inhibitors of specific DDR players, agents that interfere with hormonal signaling, or chemicals that interfere with chromatin remodeling.

DNA damaging agents. These include UV or ionizing radiation (crosslinks, dimers, and strand breaks), alkylating agents (inducing methylation), reactive oxygen species (base oxidation) [3,4], and other chemical of physical agents damaging DNA. UV and γ irradiation have been widely used on Arabidopsis [15,28,69], rice [42]), and other species. Moreover, the mechanisms of action of specific genotoxic compounds allow us to study the interactions of DDR with epigenetic and chromatin dynamics in seeds. For example, zebularine and azacytidine interfere with DNA methylation [70], whereas sodium butyrate (NaB) and trichostatin A (TSA) inhibit histone deacetylation [54,58].

DDR inhibitors. Seeds can be exposed to specific inhibitors of DDR enzymes, often consisting of molecules derived from anticancer drugs. Due to the highly conserved DDR mechanisms, the inhibitory effect is maintained in plant cells. This approach is particularly useful for inhibiting only one DDR protein to investigate alternative or redundant roles that other proteins can exert. One example is camptothecin (CPT), a well-known plant-derived inhibitor of DNA topoisomerase I that induces crosslinks and interferes with replication [60]. Similarly, another inhibitor of human tyrosyl-DNA phosphodiesterase 1 (TDP1) named NSC120686 (2-chloro-6-fluorobenzaldehyde 9H-fluoren-9-ylidenehydrazone) was used on *M. truncatula* seeds [71]. Other examples of DDR inhibitors used in seeds include zeocin (ZEO, a radiomimetic chemical that induces DSBs), curcumin (CUR, inhibitor of DSB repair and DNA damage checkpoint), and cisplatin (CIS, inducing DNA crosslinks and interfering with replication) [72]. However, despite the availability and the advantages related to this kind of molecule, the use is still limited in this context, and the possibility of specific side effects adds a layer of complexity to the interpretation of the results.

Treatments that simulate environmental stressors. Seeds are exposed to experimental systems that simulate stress conditions leading to DNA damage. Abiotic stressors such as heat, cold, drought, flooding, salinity, heavy metals, nutrient deficiency, and UV light, as well as biotic stressors like infections, pests, or herbivores, affect plant survival either by direct damage or by inducing ROS leakage from organelles (particularly mitochondria, plastids, and peroxisomes) and subsequent damage to biological macromolecules and cellular structures. This causal link between ROS accumulation and DNA damage has been reported cross-specifically during seed aging under high temperature and humidity and during imbibition/germination under a variety of environmental stressors [30,31,32].

Agents that interfere with hormonal signaling. Phytohormones can elicit genotoxic damage in seeds. For example, Arabidopsis seeds exposed to ABA displayed ROS accumulation and DNA damage [48]. Despite the efficacy of these approaches, data interpretation can be complicated by the occurrence of multiple effects. For example, prolonged exposure of *M. truncatula* seeds to kinetin anticipated germination but caused DNA damage and impaired radicle growth [73].

Chemicals that interfere with chromatin remodeling. Chromatin remodeling constitutes an essential aspect of DNA repair since histone modifications (e.g., phosphorylated histone variant γH2AX) signal DNA lesions and are involved in the recruitment of repair machinery at the damage site. Subsequently, chromatin decondensation allows the DNA repair factor to access and repair the lesion [74,75]. On the other hand, agents that induce global chromatin decondensation can expose DNA to damage. Sodium butyrate (NaB) and trichostatin A (TSA), inhibitors of histone deacetylases, have been applied to *M. truncatula* seeds [54,58]. Although such treatments are informative in investigating the interaction of DNA repair and chromatin dynamics, alteration in chromatin condensation can affect gene expression, adding a layer of complexity to data interpretation [74].

Other approaches to identify DDR hallmarks rely on GWAS (genome-wide association studies) to identify the nucleotide polymorphisms statistically associated with traits of interest. Deterioration of soybean seeds has been related to morphological features (e.g., fragile seed coat), DNA damage, oxidative damage, and ribosomal dysfunction, and QTLs (quantitative trait loci) correlated with these factors and increased seed quality in terms of longevity and vigor have been investigated [76].

Methodologically, to evaluate the use of a gene as a DDR hallmark, gene expression analyses can rely on real-time polymerase chain reaction (qRT-PCR) and high-throughput approaches relying on RNA-Seq, with novel approaches such as single-cell RNA-Seq allowing for targeted transcriptomics of specific cell subpopulations [77]. The methodological biases of transcriptomic approaches include the need to consider post-transcriptional regulation to contextualize the results and the limited availability of annotated genomes for less studies species. Moreover, the rapid turnover of transcripts during seed metabolic reactivation can represent a specific aspect to consider when applying transcriptomics to pre-germinative metabolism [44]. Despite the resource-consuming nature of gene expression analyses, they are among the most recurrent approaches for the identification of DDR-related hallmarks in pre-germinative metabolism. To reduce the methodological biases in the experimental systems, the proper experimental controls should be selected to highlight the effects of the stressors and account for possible side effects. Moreover, integration of more levels of analysis, availability of genomic resources, and development of more and more targeted transcriptomic approaches can be considered to address these methodological issues.

## 6. DDR Proteins as Hallmarks of DDR in Seeds

Gene expression profiles, based either on global transcriptomics or targeted approaches, represent a snapshot of the transcripts accumulated or depleted in a timeframe in response to specific conditions. However, due to alternative splicing and post-translational modifications, transcript quantification does not match the structural and functional variety found in the proteome [64]. Therefore, integrating transcriptomic and proteomic datasets can account for the vast array of possible variables occurring from transcripts to proteins.

Methodologically, protein separation, identification, and quantification in seeds have been achieved through a variety of approaches with different degrees of sensitivity and coverage, including gel-based approaches, liquid chromatography, mass spectrometry, and immunochemistry [78]. More recently, the high-throughput study of differentially accumulated proteins (proteomics) during germination and under stress provides an additional route for the search of DDR hallmarks. Besides the technical and economic limitations of the different proteomic approaches, a methodological limitation of using proteomics to study DDR players is the need to consider the effect of post-translational modifications on protein functionality. This is especially true when considering the signaling cascades determining DDR activation, which cannot be decoded based solely on protein abundance without considering phosphorylation.

Comparative proteomics in rice cultivars with contrastive profiles of seed storability indicated changes in the accumulation of a few protein entries associated with DNA repair, including DNA damage repair/toleration protein Os08g0129200 [79]. Another example is ITPA (inosine triphosphate pyrophosphorylase), an enzyme involved in the removal of non-canonical purine (d)NTPs inosine triphosphate, that was accumulated during imbibition in *M. truncatula* seeds [80]. Other proteomic studies suggest the role of nucleotide metabolism in support of DNA repair. Examples include AdSS (adenylosuccinate synthetase, catalyzing adenosine monophosphate biosynthesis) in *H. vulgare* and NDPK (nucleoside diphosphate kinase) in *O. sativa* and *Fagus sylvatica* [81].

Despite these protein hallmarks that are directly related to DNA damage, other processes that indirectly contribute to DNA repair can constitute sources of DDR hallmarks. For example, the PCNA protein, a key player of replication with roles in DNA repair, is responsive to fungal infections in maize [81], and the gene was upregulated in the presence of DNA damage in *M. truncatula* seeds [54]. In rice seeds, the enzymatic activity of DNA Pol λ (DNA polymerase lambda, with roles in DNA repair) is enhanced during imbibition under salt (NaCl) and osmotic (polyethylene glycol) stress [82]. Moreover, the research could be directed at proteins that exert a protective role on DNA during germination, including enzymes that detoxify ROS (a leading cause of DNA damage). Enzymatic activity quantification using colorimetric approaches was conducted in *Avena sativa* seeds subjected to aging for up to one year at difference moisture contents showing different activation patterns for SOD (superoxide dismutase), CAT (catalase) and APX (ascorbate peroxidase) enzymes [83]. Several other studies have linked antioxidant response with genome integrity in multiple phases of seed metabolism, including storage, pre-germination, seedling establishment, and stress response [30,31,32], and ROS remains a pivotal aspect to be considered when interpreting DDR activation in seeds.

LEA (late embryogenesis abundant) proteins are essential for desiccation tolerance in orthodox seeds, protecting cellular structures from dehydration and promoting metabolic inactivation through cytoplasm vitrification during storage. Despite not interacting directly with DNA, certain LEAs (e.g., LEA19A, LEA19, and LEA34) have been shown to exert a protective effect on the genome during germination [84]. Proteomic changes during lotus (*Nelumbo nucifera*) seed maturation have been studied to elucidate the acquisition of desiccation tolerance and seed longevity. A fraction of the differentially accumulated proteins were involved in DNA repair and antioxidant response, while histones, LEAs, HSPs, oligosaccharides, and ABA signaling components were enriched. Specifically, the DRT100-like (DNA-damage repair toleration) protein and the HMG (high mobility group proteins involved in chromatin structure) were accumulated during embryo maturation [85].

Concerning the post-translational modifications that have been related to DDR factors, phosphoproteomic studies highlighted the roles of protein phosphorylation in DDR signaling, starting with the phosphorylated histone variant γH2AX (involved in strand break signaling at the chromatin level) and the phosphorylation cascades initiated by the protein kinase ATM [86]. Moreover, the extensive study of protein ubiquitylation in rice seeds pinpointed DDR factors that are ubiquitylated during imbibition, including Rad23 (radiation 23) and DDI1 (DNA-damage inducible 1), involved in NER and PCNA [87].

The cited factors that show responsiveness in terms of transcriptional upregulation and/or protein accumulation to experimental conditions tested in seeds are summarized in Table 1 (entries involved in DDR sensing and signaling), Table 2 (entries involved in DNA repair), and Table 3 (entries interacting with DDR as transcriptional factors, splicing factors, miRNA or rRNA).

## 7. Chromatin Remodeling and Epigenetic Modifications Influencing Seed DDR

Chromatin dynamics constitute a fundamental regulatory aspect of replication, transcription, and recombination by modulating DNA accessibility and factor recruitment, with relevant implications for DDR signaling, DNA repair, cell cycle progression, endoreduplication, PCD, and maintenance of genome stability [90]. In eukaryotes, DDR is initiated at the chromatin level and is strongly influenced by chromatin condensation status, histone modifications (e.g., acetylation, deacetylation, and substitution), epigenetic regulation (DNA methylation and demethylation), and other factors, such as noncoding RNAs [74]. Methodologically, histone modifications and DNA methylation can be detected using immunochemical approaches, whereas high-throughput methylomic approaches (such as whole-genome bisulfite sequencing) can map DNA methylation genome-wide [12,74,90].

Proteins involved in chromatin remodeling, particularly histones, are promising candidates as DDR hallmarks. Histone variants, such as the phosphorylated histone γH2AX, are involved in ATM-dependent DSB signaling and recruitment of DNA repair machinery [5,12], with functions highlighted in *M. truncatula* seeds responding to rehydration-dehydration cycles inducing DNA damage and ROS accumulation [49]. The decreased accumulation of histone proteins, as well as other proteins involved in DNA packing and chromatin processes, was linked to germination failure in *H. vulgare* seeds [91]. On the other hand, the gene encoding histone H2B was upregulated in maize seeds in response to camptothecin [60].

Factors operating at the interface between chromatin remodeling and DDR can represent a source of highly specific DDR hallmarks, as in the case of protein kinase CK2 required for genome maintenance in plants. Specifically, Arabidopsis *ck2* mutants are hypersensitive to multiple types of DNA lesions induced by γ irradiation and display higher occurrence of DNA damage signaling via phosphorylated histone variants, chromatin decondensation, and non-conservative DNA repair mechanisms [69].

The nucleosomal organization is regulated through ATP-dependent chromatin remodelers (ACRs), chromatin modifiers, and histone chaperones. ACR also contributes to homolog recombination DNA repair under genotoxic stress [75], while other factors are involved in chromatin remodeling and DDR in seed metabolism. Examples include HAM1 and HAM2 (histone acetyltransferase of the MYST family, with roles in seed development and DNA repair following UV-B damage) and NRP1 (NAP (nucleosome assembly protein)-related protein, a histone chaperone involved in the recruitment of DNA repair enzymes) [75]. Moreover, TRRAP (transformation/transactivation domain-associated protein) acts as a scaffold for the assembly of HAT (histone acetyltransferase) complexes to allow chromatin remodeling during DNA repair in seeds. Consistently, the TRRAP gene is upregulated in *M. truncatula* seeds and seedlings challenged with the histone deacetylase inhibitor trichostatin A, in association with DNA damage, an upregulation of DDR and antioxidant genes, and an accumulation of antioxidant compounds [58]. NSE4A (non-SMC element 4A), a subunit of the SMC5/6 (structural maintenance of chromosome 5/6) complex involved in chromosome condensation, has roles in seed development and accumulates during DNA repair in meristems. Arabidopsis *nse4a* mutants are hypersensitive to zebularine and develop seeds with cellular and morphological abnormalities [70].

Along with various functions in gene expression regulation, cell growth and proliferation, and stress response, DNA methylation in seeds is also involved in preserving DNA against non-specific endonuclease cutting and transposon-mediated recombination events [81]. Changes in DNA methylation were observed in γ irradiated Arabidopsis and radio-contaminated soybean seedlings [74], and the correlation between DNA methylation patterns and DDR is still under investigation. For instance, histone H2AX promotes DNA methylation in Arabidopsis seed endosperm; *h2ax* mutant seeds were more sensitive to genotoxic stress and displayed lower GC methylation in transposons, suggesting protective mechanisms against transposon-mediated recombination [92]. In sycamore (*A. pseudoplatanus*) seeds subjected to desiccation stress and artificial aging, changes in the global levels of m5C, hm5C, and 8-oxoG (8-oxo-7,8-dihydroguanine) were observed in association with ROS accumulation and reduced viability. Particularly, a decrease in m5C was observed, consistently with other studies performed in aging oak (*Quercus robur*) seeds [53,93].

DNA methylation patterns and epigenetic signatures can be maintained in time and across generations, allowing for stress memory mechanisms. This applies to the positive effects of seed priming, including enhanced DNA repair ability, which can be maintained after priming and transmitted to the offspring [94]. Such findings underline the relevance of these mechanisms, envisaged as a promising direction to investigate stress tolerance.

A summary of the cited chromatin remodeling factors with roles in DNA repair is provided in Table 4, including entries that show responsiveness (transcriptional upregulation, protein accumulation in seeds) to the tested experimental conditions.

## 8. Seed Metabolites and Small Molecules as DNA Damage Hallmarks

Metabolomic approaches constitute another promising level of analysis to identify molecular signatures of stress tolerance and possible directions for crop improvement, especially thanks to the biochemical and functional heterogeneity of target molecules that can be considered as universal or specific hallmarks of seed quality. Methodologically, a majority of plant metabolomic studies rely on liquid chromatography (LC) or gas chromatography (GC) coupled with mass spectrometry (MS), with a variety of columns allowing for the separation of amino-acids, oligosaccharides, phytohormones, and other plant metabolites that require specific analytic approaches based on their biochemical nature. Moreover, the heterogeneity of plant metabolites and the presence of volatile compounds may require derivatization steps to increase resolution and sensitivity. In this sense, targeted approaches focusing on a specific class of metabolites might be preferred over non-targeted approaches providing a comprehensive metabolomic evaluation [95].

Several studies have summarized the metabolomic changes occurring in seeds and seedlings responding to heat and drought stress in crop species such as *O. sativa*, *G. max*, *H. vulgare*, and *Vigna unguiculata* [95]. Nonetheless, the instances directly linking differential metabolite accumulation with DNA damage or DDR are still rare.

Metabolic signatures of DNA degradation were found in *M. truncatula* seeds in the presence of DNA damage and impaired seedling development. These included 3-UPA (3-ureidopropionate, uracil metabolism), 5-methylcytidine and N2, N2-dimethylguanosine (related with tRNA modifications participating in the post-transcriptional regulation of DDR), and xanthine (intermediate of purine degradation) [54,73]. In aging sunflower (*Helianthus annuus*) seeds, DNA fragmentation, ROS accumulation, and impaired germination were accompanied by significant changes in the adenylate (ATP, ADP, and AMP) pool [96]. On the other hand, differential accumulation of metabolites with DNA protective functions can be indicative of DNA damage status, especially considering the relevance of antioxidant mechanisms in stored, aging, and germinating seeds. Differential accumulation of the polyamine agmatine, along with the overexpression of polyamine metabolism genes, was highlighted in *M. truncatula* seeds in the presence of DNA damage [54]. Spermidine was also tested as a vigorization agent on desiccation-sensitive *Acer saccharinum* seeds, with beneficial effects on DNA integrity, antioxidant response, and germination rates [97]. In this sense, metabolites enhancing DNA integrity are particularly promising as bioactive compounds to be administered in seed vigorization protocols.

Molecules involved in signaling pathways interacting with DDR can also be considered among the possible stress-responsive DDR hallmarks of seed metabolism. Retrograde signaling pathways, allowing communication between organelles, rely on small molecules such as PAP (3′-phosphoadenosine 5′-phosphate), which is involved in the plastid, mitochondria, and nucleus retrograde signaling [98]. PAP was strongly depleted in *M. truncatula* seeds under dehydration stress, in association with increased DNA damage, higher ROS levels, and DDR activation [49].

## 9. Conclusions and Future Perspectives

This review has reported the current efforts to identify the molecular players related to DDR in seed metabolism, proposing possible criteria and constraints for their application as hallmarks of DNA damage and repair in seeds. The strain for universality is currently limited by the lack of systematic evaluations accounting for inter- and intra-specific differences in a wide array of model and crop species. Increases in reliability will require a deeper and more integrative understanding of the molecular processes involved in stress response in the wider context of seed and seedling metabolism. This could be achieved through targeted experimental systems along with principles of systems biology [44]. This aspect is linked to the ability to provide mechanistic explanations to represent the complexity of observed responses in consistent models. In silico models can contextualize the reliability of a given hallmark across a variety of dimensions, such as activation timeframes and stress specificity. As universality and reliability are progressively addressed, the technical constraints of detectability need to be overcome to allow for a rapid and precise assessment of the seed stress status. Despite these challenges, it is reasonable to speculate that metabolites related to genotoxic stress can constitute hallmarks with high detectability profiles (e.g., as volatile compounds) for stress fingerprinting or as proxies of seed deterioration and that compounds preserving DNA integrity can be implemented in seed priming protocols or seed coating formulations [1,2,47]. Developments in these directions would benefit the current management of germplasm resources. Moreover, key genes of DDR and stress response can be considered for biotechnological approaches or marker-assisted breeding programs toward climate-ready crops and resource-efficient agrosystems [2,44]. A novel theoretical framework in different experimental systems is necessary to elucidate the implications and interactions of specific hallmarks in different crops, thus providing seed operators (biotechnologists, breeders, industries, and farmers) with versatile or targeted solutions to be tested and implemented [44]. In a broader perspective, DDR-related hallmarks in seed metabolism contribute to an expanding list of intervention options to express the full potential of seed resources toward food security and sustainability.

## Figures and Tables

**Table 1:** Summary of the putative molecular hallmarks (genes, proteins) related to DDR sensing, transduction, and regulation with reported responsiveness to the experimental conditions tested in seeds.

Gene/Protein	Annotated Function (s)	Responsiveness in Seed Systems	Species	Reference
MRN (MRE11, RAD50, NBS1)	DDR DSB sensing	ABA, ROS, DNA damage	*A. thaliana*	[48]
		Hydropriming and desiccation	*M. truncatula*	[49]
		CPT, DNA damage	*M. truncatula*	[71]
		NaCl	*S. melongena*	[55]
ATM	DDR signaling	Aging	*A. thaliana*	[31]
ATR	DDR signaling	Aging	*A. thaliana*	[31]
		γ-irradiation	*O. sativa*	[42]
SMR	Kinases activated by ATM	Repair after aging, germination delay in the presence of damage	*A. thaliana*	[31,32]
SOG1	DDR transcriptional activation, regulation of cell cycle, endoreduplication, PCD	γ-irradiation	*A. thaliana*	[12]
		Induction of PCD after aging	*A. thaliana*	[32]
		Root growth in the presence of DNA lesions	*A. thaliana*	[50]
		Imbibition, hydropriming, overpriming	*M. truncatula*	[16,49]
		CPT, DNA damage	*M. truncatula*	[71]

**Table 2:** Summary of the putative molecular hallmarks (genes, proteins) involved in DNA repair with reported responsiveness to the experimental conditions tested in seeds.

Gene/Protein	Annotated Function (s)	Responsiveness in Seed Systems	Species	Reference
OGG1	BER, repair of 7,8-dihydro-8-oxoguanine	Methyl viologen, NaCl, and mannitol associated with longevity	*A. thaliana*	[38]
		Imbibition	*G. max*	[39]
		Imbibition	*M. truncatula*	[37]
		TSA, DNA damage	*M. truncatula*	[58]
		NaB, DNA damage	*M. truncatula*	[54]
		NaCl	*S. melongena*	[55]
		Hydropriming	*S. melongena*	[45]
		Imbibition	*T. aestivum*	[40]
		Imbibition	*H. vulgare*	[40]
		Imbibition	*S. cereale*	[40]
FPG	BER, repair of formamido-pyrimidine	Imbibition	*M. truncatula*	[37]
		NaB, DNA damage	*M. truncatula*	[54]
		Hydropriming	*S. melongena*	[45]
		Imbibition	*T. aestivum*	[40]
		Imbibition	*H. vulgare*	[40]
		Imbibition	*S. cereale*	[40]
MBD4L	BER, DNA glycosylase	Imbibition in aged seeds and in mutants displaying impaired germination	*A. thaliana*	[57]
ALKBH1	BER, removal of alkylated bases	NaB, DNA damage	*M. truncatula*	[54]
RPh16	NER	CPT	*Z. mays*	[60]
MtERCC1	NER	CPT, DNA damage	*M. truncatula*	[71]
UDG	base-excision in mitochondria	γ-irradiation	*O. sativa*	[42]
RAD51-54	HR	ABA-induced DNA damage	*A. thaliana*	[48]
		CPT, DSBs, and cell death	*Z. mays*	[60]
BRCA1	HR	ABA-induced DNA damage	*A. thaliana*	[48]
XRI-1	HR	CPT	*Z. mays*	[60]
LIG1	NHEJ	Imbibition	*A. thaliana*	[38]
LIGIV	NHEJ	ABA, ROS, DNA damage	*A. thaliana*	[48]
		TSA, DNA damage	*M. truncatula*	[58]
Ku70-80	NHEJ	ABA, ROS, DNA damage	*A. thaliana*	[48]
		NaCl	*S. melongena*	[55]
XRCC4	NHEJ	ABA, ROS, DNA damage	*A. thaliana*	[48]
PARP	Ku-independent NHEJ	γ-irradiation	*O. sativa*	[42]
		CPT, DNA damage	*M. truncatula*	[71]
MSH2	MMR	NaCl	*S. melongena*	[55]
PCNA	Replication, DNA repair recruitment	NaCl	*S. melongena*	[55]
		NaB, DNA damage	*M. truncatula*	[54]
Pol Lambda	DNA polymerase, DNA repair	NaCl, PEG	*O. sativa*	[81]
RecA	Recombination	γ-irradiation	*O. sativa*	[42]
Tdp1	DNA-topoisomerase crosslinks	TSA, DNA damage	*M. truncatula*	[58]
		NSC, DNA damage	*M. truncatula*	[71]
		PEG, DNA damage	*M. truncatula*	[88]
TFIIS	Prevention of damage during transcription	PEG DNA damage	*M. truncatula*	[89]
RNR1	Providing dNTPs for DNA repair	CPT	*Z. mays*	[60]
Rpa2	ssDNA binding in repair and recombination	CPT	*Z. mays*	[60]
MUS81	Resolution of DNA junctions	CPT, DNA damage	*M. truncatula*	[71]

**Table 3:** Summary of the putative molecular hallmarks interacting with DDR as transcriptional factors, splicing factors, rRNA, and miRNA with reported responsiveness to the experimental conditions tested in seeds.

Gene/Protein	Annotated Function (s)	Responsiveness in Seed Systems	Species	Reference
OFP	TF involved in DNA repair, ROS homeostasis, response to GA and ABA	Germination	*A. thaliana*	[61]
NAC103	TF regulating ABA-responsive DDR genes	Seed and seedling growth	*A. thaliana*	[62]
ABI3	ABA sensitivity, seed dormancy, and longevity	Dormancy and longevity	*A. thaliana*	[33]
TERF1	DDR gene repressor	Germination in darkness	*N. tabacum*	[63]
DRT111	Splicing factor of ABA sensitivity genes	Negative regulation of ABA sensitivity in germination	*A. thaliana*	[65]
rRNA 5.8S	rRNA	Hydropriming and dehydration, ROS, and DNA damage	*M. truncatula*, *M. sativa*	[49]
tae-miR5086	miRNA targeting RAD50 transcript	γ-irradiation	*T. aestivum*	[66]

**Table 4:** Summary of the putative molecular hallmarks (genes, proteins) related to chromatin remodeling with functions in DDR with reported responsiveness to experimental conditions tested in seeds.

Gene/Protein	Annotated Function (s)	Responsiveness in Seed Systems	Species	Reference
γH2AX	Histone, DSB signaling	Hydropriming, dehydration, DNA damage, ROS	*M. truncatula*	[49]
H2B	Histone involved in DNA repair	CPT	*Z. mays*	[60]
H2A6, H2A, 2B3, H4	Histones	Germination	*H. vulgare*	[90]
DRT100-like	DNA-damage repair toleration, chromatin structure	Embryo maturation, longevity	*N. nucifera*	[85]
HAM1, HAM2	Histone acetyltransferase, DNA repair	Seed development, UV-B damage	*A. thaliana*	[75]
TRRAP	Scaffold in histone acetyltransferase and DNA repair complexes	TSA, DNA damage	*M. truncatula*	[58]
NSE4A	Chromosome condensation	DNA repair in meristems, zebularine hypersensitivity	*A. thaliana*	[70]

## Data Availability

Bibliographic references are provided for the data exposed in this review.
